# Students' self-regulated learning (SRL) profile dataset measured during Covid-19 mitigation in Yogyakarta, Indonesia

**DOI:** 10.1016/j.dib.2020.106422

**Published:** 2020-10-20

**Authors:** Dwi Sulisworo, Meita Fitrianawati, Ika Maryani, Saleh Hidayat, Erie Agusta, Wulandari Saputri

**Affiliations:** a Ahmad Dahlan University, Indonesia; b Muhammadiyah University of Palembang, Indonesia

**Keywords:** Self-regulated learning, Education policy, Online learning, Covid-19, Learning achievement

## Abstract

The Covid-19 pandemic has made changes in various sectors of life in Indonesia, including education. The Indonesian Ministry of Education and Culture issued a policy for the implementation of online learning. One of the factors that determine the success of online learning is the level of student self-regulated learning. Thus understanding the capabilities of SRL is essential for achieving successful education during this pandemic. This article presents data that explore the profiles of self-regulated learning in 1st-grade to 12th-grade students. Four aspects of self-regulated learning include planning, monitoring, controlling, and reflecting. Data retrieval is related to predictions of online learning success during Covid-19 mitigation. The sample consisted of 6571 students. The questionnaire was distributed to 61 schools (37 primary schools, 12 junior high schools, and 12 senior high schools) with an online survey in Yogyakarta, Indonesia. The questionnaire was prepared in an online format using Google Form. This link was presented with an introductory sentence from the researcher and distributed to students through the respective principal. Students may only fill in a questionnaire once but were allowed to make changes in response. The collected data were selected to be valid and reliable using the Rasch model. Some data released are items that are not filled in, extremely low or high data. These data can be further processed with various statistical techniques such as Two-way, ANOVA, MANOVA, or Cluster Analysis following the intended in-depth analysis needs. The data will be useful for researchers, educational decision-makers, and education managers to improve online learning services and implementation that enhance student learning achievement.

## Specifications Table

**Table:** 

Subject	Education
Specific subject area	Educational Psychology, especially on self-regulated learning profiles
Type of data	Table
How the data were acquired	Survey with an online questionnaire (see Table 2)
Data format	Raw Analyzed
Parameters for data collection	Students from 1st-grade 1 to 12th-grade participating in online learning during Covid-19 mitigation at Muhammadiyah schools in Yogyakarta, Indonesia.
Description of data collection	The questionnaire was for obtaining aspects of the SRL (planning: 5 items, monitoring: 6 items, controlling: 6 items, and reflecting: 5 items) of the students from 1st-grade to 12th-grade. The statements were in the form of positive and negative statements using a Likert scale ranging from score 1 (strongly disagree) to 5 (strongly agree). The questionnaire was prepared in an online format using Google Form. This questionnaire link was presented with an introductory sentence from the researcher and was distributed to students through the respective principal. Students may only fill in a questionnaire once but were allowed to make changes in response. The collected data were selected to be valid and reliable using the Rasch model. Some data released are items that are not filled in, extremely low or high data. The data collection permit was obtained from the Basic and Secondary Education Assembly, Regional Leader of Muhammadiyah, Yogyakarta City. The data were taken in the period 18–25 March 2020.
Location source data	Institution: Muhammadiyah Schools City: Yogyakarta Country: Indonesia Latitude and longitude for collected samples/data: (-7.803164, 110.3398253) https://goo.gl/maps/Ps6HtDXjYsaax5G4A
Accessibility data	Repository name: http://eprints.uad.ac.id/ Data identification number: - Direct URL to data: http://eprints.uad.ac.id/18871/1/Supplementary%20Data.xlsx (Supplementary data)

## Value of the Data

• This data are useful to understand students' SRL profiles as a basis for predicting learning success.
• More specifically, these data can be useful for predicting online learning success during Covid-19 mitigation.
• Other researcher can process this data with various statistical techniques such as Two-way, ANOVA, MANOVA, or Cluster Analysis following the intended in-depth analysis needs.
• The data will be useful for researchers, educational decision-makers, and education managers to improve online learning services and implementation that enhance student learning achievement.
• The method used in collecting these data can be replicated in other regions and make SRL comparisons between them.

## 1 Data Description

This dataset contains SRL data on four factors (Planning, Monitoring, Controlling, and Reflecting) with the item structure, as presented in Table 2. Item details for each factor are shown in Table 3. Based on these items, a questionnaire was developed to measure the SRL. The questionnaire was distributed to 61 schools (37 primary schools, 12 junior high schools, and 12 senior high schools) with an online survey. The raw data obtained from student responses can be seen in Table 1S (See: Supplementary Data). In Table 1S, all scores have considered negative statements.

Furthermore, these data were processed by the Rasch Model; they were displayed in Table 2S (See: Supplementary Data). From this analysis, data fit (Table 3S) was obtained, it can be used for various purposes (See: Supplementary Data). The primary processing done calculates the Mean, Standard Deviation for each aspect of Self-regulated learning (Table 4S; See: Supplementary Data). The extract is in Table 1, representing the descriptive of SRL aspects. Each item of the questionnaire's detailed response is provided as a supplementary file (see the link as mentioned in the ‘Accessibility data’ section).

**Table 1:** Descriptive of SLR aspects.

Items	Strongly Disagree (1)	Disagree (2)	Neutral (3)	Agree (4)	Strongly Agree (5)	Mean	Standard Deviation	N
P1	68	146	682	1094	1866	4.178431	0.966953	3874
P2	120	301	972	1124	1352	3.849574	1.080357	3874
P3	59	141	624	1073	1965	4,228,384	0.948257	3874
P4	168	408	1053	1118	1117	3.674935	1.125455	3874
P5	473	721	1137	907	623	3.125888	1.239552	3874
M1	94	248	990	1289	1239	3,862,943	1.015565	3874
M2	139	250	878	1272	1317	3.876056	1.064259	3874
M3	76	235	1019	1238	1288	3,888,751	1.001325	3874
M4	144	301	901	1286	1230	3,81,746	1.078186	3874
M5	695	888	1081	634	561	2.864713	1.293032	3874
M6	402	632	1061	930	836	3.301988	1.261341	3874
C1	72	178	865	1258	1484	4,012176	0.978523	3874
C2	107	204	914	1200	1436	3.946399	1.031132	3874
C3	34	86	638	1149	1953	4.26969	0.87426	3874
C4	29	74	423	1059	2277	4.419215	0.81591	3874
C5	876	967	1098	581	341	2.623082	1.230814	3874
C6	294	378	690	861	1639	3.82159	1.28187	3874
R1	48	163	997	1422	1234	3,939,701	0.9232	3874
R2	53	121	454	913	2326	4,380,398	0.906333	3874
R3	55	151	966	1309	1382	3,986,807	0.94331	3874
R4	47	90	444	983	2301	4.397419	0.867935	3874
R5	214	263	612	853	1926	4,037749	1.19281	3874

## 2 Experimental Design, Materials and Methods

This data gathering was ex-post facto design. The data were taken by a survey using an SRL questionnaire. The SRL model used refers to [1] with a questionnaire structure like Table 2.

**Table 2:** Questionnaire structure.

		Item number		
No	Factors	Positive Statements	Negative Statements	Total
1	Planning	1, 2, 3, 4	5	5
2	Monitoring	6, 7, 8, 9, 10	11	6
3	Controlling	12, 13, 14, 15	16, 17	6
4	Reflecting	18, 19, 20, 21	22	5

Table 3 shows the statement of each factor. Each statement of the questionnaire items was adapted from [1]. This questionnaire was translated into Indonesian and validated before used.

**Table 3:** Items of the questionnaire.

General statements	Item Code
Plan	
1. I plan out projects that I want to complete.	P1
2. If an important test is coming up, I created a study plan.	P2
3. Before I do something fun, I consider all the things that I need to get done.	P3
4. I can usually estimate how much time my homework will take to complete.	P4
5. I have trouble making plans to help me reach my goals. (N)	P5
Monitor	
6. I keep track of how my projects are going.	M1
7. I know when I'm behind on a project.	M2
8. I track my progress in reaching my goals.	M3
9. I know what my grades are at any given time.	M4
10. Daily, I identify things I need to get done and track what gets done.	M5
11. I have trouble remembering all the things I need to accomplish. (N)	M6
Control	
12. I do what it takes to get my homework done on time.	C1
13. I make choices to help me succeed, even when they aren't the most fun right now.	C2
14. As soon as I see things aren't going right, I want to do something about it.	C3
15. I keep trying as many different possibilities as necessary to succeed.	C4
16. I have difficulty maintaining my focus on projects that take a long time to complete. (N)	C5
17. When I get behind on my work, I often give up. (N)	C6
Reflect	
18. I think about how well I'm doing on my assignments.	R1
19. I feel a sense of accomplishment when I get everything done on time.	R2
20. I think about how well I've done in the past when I set new goals.	R3
21. When I fail at something, I try to learn from my mistake.	R4
22. I keep making the same mistake over and over again. (N)	R5

The method for obtaining data that fit is using the Rasch model [2,3]. Some fit indexes provided in the Rasch analysis are ZSTD Person Infit, ZSTD Person Outfit, MNSQ Infit Person, MNSQ Person Outfit, ZSTD Infit Item, ZSTD Outfit Item, MNSQ Infit Item, MNSQ Outfit Item [2]. The data from the raw data (Table S1; look at supplementary data) were processed using the WINSTEP 3.73 application to obtain pertinent data. The data were processed with the Rasch model (Table S2; Look at supplementary data). From 6613 out of total respondents were analyzed. This action was conducted because 46 respondents filled in their identity but did not fill in the questionnaire. Therefore, there were 6659 – 46 = 6613 respondents only (see Table S2, Supplementary Data), the respondents who met the criteria.

1. Outfit Mean Square (MNSQ) received: 0.5 <MNSQ <1.5
2. Z-Standard Outfit (ZSTD) value received: –2.0 <ZSTD <+2.0
3. Outfit Point Value Correlation (Pt Mean Corr) Value: 0.4 <Pt Measure Corr <0.85.

So if fit data are collected, there are 3874 fit total respondents. The rest of the respondents who did not fit were 2785 people consisting of 42 people indicated as outliers (40 people from the maximum extreme score group and two people from the minimum extreme score group), 46 people were indicated not filling in the questionnaire. However, they had identities, and 2697 entered the most misfitting responses and the most unexpected responses.

## Ethics Statement

Before the participant filled in the questionnaire, he/ she had to give his/ her informed consent. If he/ she did not provide it, he/ she would automatically leave from the application. Therefore, all the datasets were obtained with the proper procedure, including the participant's informed consent.

## Declaration of Competing Interest

The authors declare that they have no conflict of interest that could influence this work.

## Appendix Supplementary materials

Image, application 1
